# C-Peptide Increases Na,K-ATPase Expression via PKC- and MAP Kinase-Dependent Activation of Transcription Factor ZEB in Human Renal Tubular Cells

**DOI:** 10.1371/journal.pone.0028294

**Published:** 2011-12-05

**Authors:** Dana Galuska, Sergej Pirkmajer, Romain Barrès, Karin Ekberg, John Wahren, Alexander V. Chibalin

**Affiliations:** 1 Section of Integrative Physiology, Department of Physiology and Pharmacology, Karolinska Institutet, Stockholm, Sweden; 2 Section of Integrative Physiology, Department of Molecular Medicine and Surgery, Karolinska Institutet, Stockholm, Sweden; Université Joseph Fourier, France

## Abstract

**Background:**

Replacement of proinsulin C-peptide in type 1 diabetes ameliorates nerve and kidney dysfunction, conditions which are associated with a decrease in Na,K-ATPase activity. We determined the molecular mechanism by which long term exposure to C-peptide stimulates Na,K-ATPase expression and activity in primary human renal tubular cells (HRTC) in control and hyperglycemic conditions.

**Methodology/Principal Findings:**

HRTC were cultured from the outer cortex obtained from patients undergoing elective nephrectomy. Ouabain-sensitive rubidium (^86^Rb^+^) uptake and Na,K-ATPase activity were determined. Abundance of Na,K-ATPase was determined by Western blotting in intact cells or isolated basolateral membranes (BLM). DNA binding activity was determined by electrical mobility shift assay (EMSA). Culturing of HRTCs for 5 days with 1 nM, but not 10 nM of human C-peptide leads to increase in Na,K-ATPase α_1_-subunit protein expression, accompanied with increase in ^86^Rb^+^ uptake, both in normal- and hyperglycemic conditions. Na,K-ATPase α_1_-subunit expression and Na,K-ATPase activity were reduced in BLM isolated from cells cultured in presence of high glucose. Exposure to1 nM, but not 10 nM of C-peptide increased PKCε phosphorylation as well as phosphorylation and abundance of nuclear ERK1/2 regardless of glucose concentration. Exposure to 1 nM of C-peptide increased DNA binding activity of transcription factor ZEB (AREB6), concomitant with Na,K-ATPase α_1_-subunit mRNA expression. Effects of 1 nM C-peptide on Na,K-ATPase α_1_-subunit expression and/or ZEB DNA binding activity in HRTC were abolished by incubation with PKC or MEK1/2 inhibitors and ZEB siRNA silencing.

**Conclusions/Significance:**

Despite activation of ERK1/2 and PKC by hyperglycemia, a distinct pool of PKCs and ERK1/2 is involved in regulation of Na,K-ATPase expression and activity by C-peptide. Most likely C-peptide stimulates sodium pump expression via activation of ZEB, a transcription factor that has not been previously implicated in C-peptide-mediated signaling. Importantly, only physiological concentrations of C-peptide elicit this effect.

## Introduction

C-peptide, the connecting segment of proinsulin, is secreted by pancreatic β-cells into the circulation together with insulin in equimolar quantities. One role of C-peptide is to participate in the proper folding of proinsulin by facilitating correct disulfide bond formation between the A- and B-chain of insulin. A series of studies during the past decade have presented new aspects of C-peptide physiology. C-peptide administration corrects glomerular hyperfiltration characteristic of the early stages of diabetic nephropathy, reduces urinary excretion of albumin and prevents the development of glomerular hypertrophy in type 1 diabetes [1]. C-peptide infusion prevents experimentally induced type 1 diabetes-dependent decrease of renal Na,K-ATPase α_1_-subunit in rats [2]. In patients with type 1 diabetes and in animal models of the disease, administration of C-peptide in physiological concentrations results in improvements of diabetes-induced functional and structural changes of peripheral nerves [3], [4], [5]. C-peptide in replacement doses prevents diabetes-induced deficits in nerve fiber regeneration [6], protects against glucose-induced apoptosis, and stimulates cellular proliferation [7].

The molecular mechanisms by which C-peptide exerts its effects are now beginning to emerge. C-peptide binds to membrane binding site on a number of different cell types, thereby triggering pertussis toxin sensitive, Ca^2+^-dependent intracellular signaling pathways, including protein kinase C (PKC) isoforms and the mitogen-activated protein (MAP) kinase cascade [8], [9]. C-peptide may exert insulinomimetic effects via interaction with the insulin signaling pathways at the level of the insulin receptor or downstream of it [10]. C-peptide acutely stimulates Na,K-ATPase activity via PKC and MAP activation kinase activation in human renal tubular cells [11], [12]. Activation of the Na-pump is of particular clinical interest, as it is reported to be deficient in type 1 diabetes in a number of tissues [2], [13], [14]. Given the central role of Na,K-ATPase in the regulation of intracellular ion concentrations, a reduction in Na,K-ATPase activity may contribute to decreased nerve conduction velocity, retinal cell dysfunction, impaired endothelial function and decreased microvascular blood flow, kidney disorders and development of hyperkalemia [15], [16], [17]. The decreased Na,K-ATPase activity found in association with diabetes mellitus and its complications can be restored to normal by administration of C-peptide [2], [3], [18], although the mechanism of this stimulation is not completely understood.

Na,K-ATPase is a ubiquitously expressed plasma membrane cation pump, which is essential for maintenance of intracellular and extracellular sodium and potassium concentrations, cell volume, osmotic balance and electrochemical gradients [19]. The regulation of Na,K-ATPase can be achieved via multiple mechanisms, including changes in intrinsic activity, subcellular distribution and cellular abundance [20]. Apart from classical regulation by steroid and thyroid hormones [20], the molecular regulation of genes encoding Na,K-ATPase subunits under physiological conditions where Na,K-ATPase expression is altered, such as hypokalemia [21], starvation [22], or diabetes [13], is largely unknown. The gene promoter of the Na,K-ATPase α_1_-subunit predominantly expressed in the kidney contains consensus sequences for several transcription factors [23]. Moreover, MAP kinases have been implicated in the regulation of Na,K-ATPase expression. Activation of ERK1/2 signaling pathway leads to an increase in synthesis of Na,K-ATPase subunits [24]. Consequently, in this study we examined the molecular mechanism by which long term exposure to physiological and elevated concentration of C-peptide stimulates Na,K-ATPase expression and activity in primary human renal tubular cells in control and hyperglycemic conditions.

## Results

### Physiological concentrations of C-peptide stimulate Na,K-ATPase protein expression and activity in HRTC in culture

**Figure 1:**
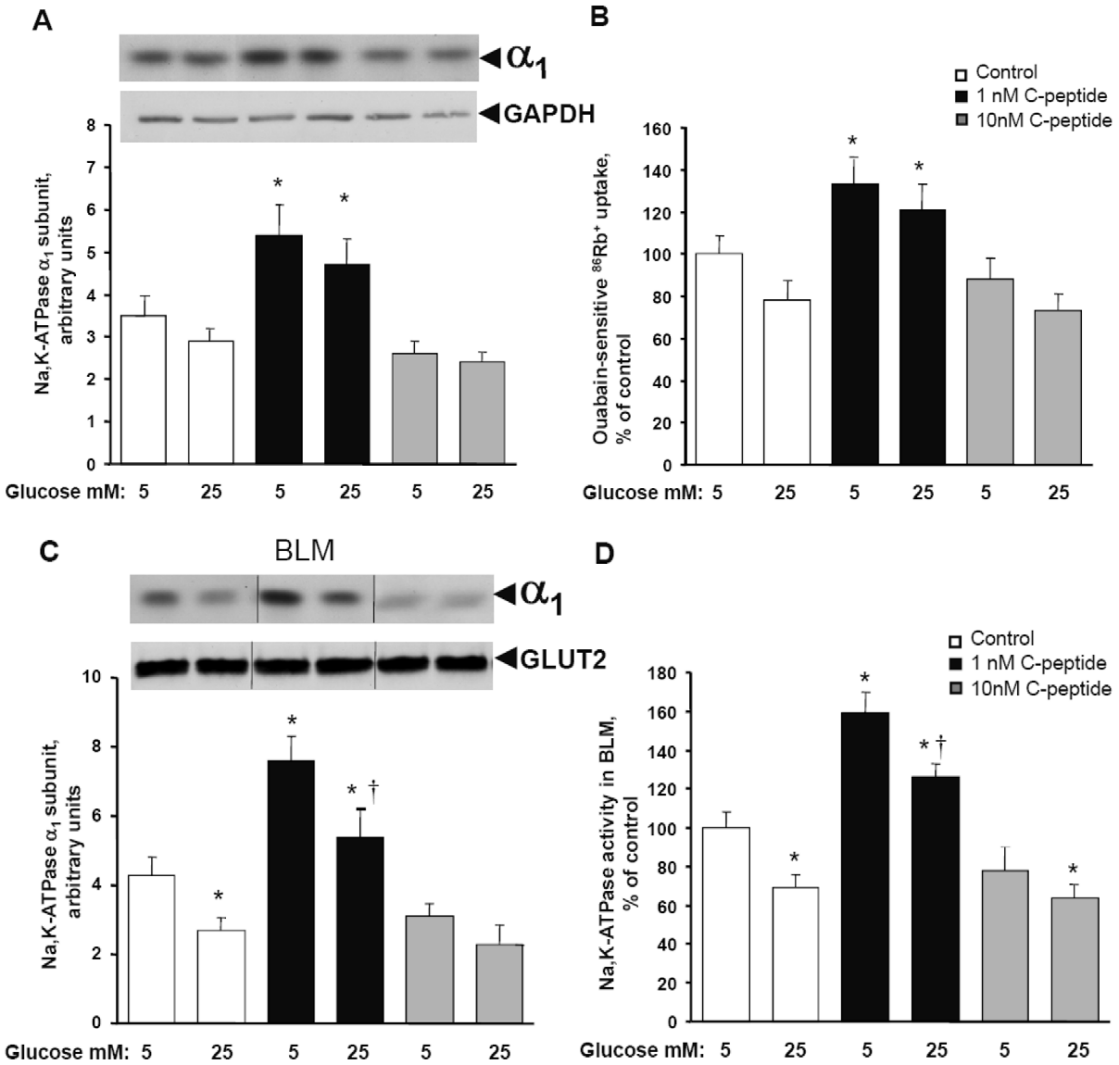
Effects of different C-peptide and glucose concentrations on Na,K-ATPase expression and activity in HRTC. Cells were cultured and analyzed as described in “Materials and Methods”. **A.** C-peptide at 1 nM concentration stimulates Na,K-ATPase protein expression. A representative Western blot image is shown in the upper panel. GAPDH protein was used as a loading control. **B.** Ouabain-sensitive ^86^Rb^+^ uptake in intact HRTC. **C.** Na,K-ATPase α_1_-subunit abundance in basolateral membrane fractions isolated from HRTC. A representative Western blot image is shown in the upper panel. GLUT2 protein was used as a loading control. **D.** Na,K-ATPase enzymatic activity in basolateral membrane fractions isolated from HRTC. Results are means ± SE for 6 independent experiments. * P<0.05 versus 5 mM glucose without C-peptide. † P<0.05 versus 5 mM glucose with 1 nM of C-peptide.

Culturing of HRTCs with 1 nM, but not 10 nM, of human C-peptide for 5 days leads to an increase in Na,K-ATPase α_1_-subunit protein expression (Fig. 1A), accompanied with increase in Na,K-ATPase ion transporting activity measured as ^86^Rb^+^ uptake (Fig. 1B), both in normal- and hyperglycemic conditions. Scrambled C peptide has no effect on Na,K-ATPase expression and activity (data not shown). Viability and morphology as assessed by light microscopy of HRTC after incubation with the C-peptide were similar to those for control cells. The Na,K-ATPase α_1_-subunit abundance and Na,K-ATPase enzymatic activity were also increased in basolateral membrane fractions isolated from HRTC (Fig. 1C and D). While high glucose concentration does not affect Na,K-ATPase α_1_-subunit expression and Na,K-ATPase activity when determined in whole cells, Na,K-ATPase α_1_-subunit abundance (Fig. 1C) and Na,K-ATPase enzymatic activity (Fig. 1D) were specifically reduced in basolateral membrane fractions isolated from cells cultured in presence of high glucose, suggesting that during hyperglycemia Na,K-ATPase internalized from cell surface and sequestered in an intracellular stores.

### Effects of long term C-peptide exposure on PKC activation

**Figure 2:**
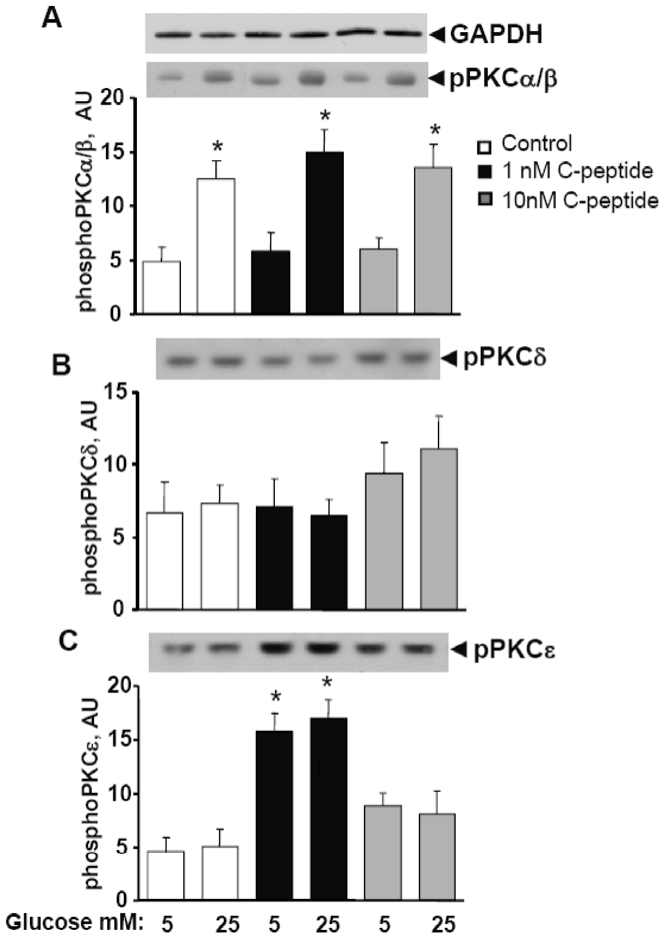
Culturing of HRTCs with 1 nM of C-peptide resulted in increase in PKCε phosphorylation, while phosphorylation of PKCs α/β increased by high glucose concentrations. Cells were cultured as described in “Materials and Methods”. Total cell lysates were subject to Western blot analysis to determine PKCs phosphorylation. A representative Western blot image is shown in the upper panel of each graph. **A.** Phospho PKCα/β. **B.** Phospho PKCδ, **C.** Phospho PKCε. GAPDH protein was used as a loading control. Results are means ± SE for 6 independent experiments. * P<0.05 versus 5 mM glucose without C-peptide.

Acute stimulation by C peptide specifically activates PKC isoforms δ and ε in HRTC [8]. Phospho-PKC isoform-specific antibodies (pPKC α/β, δ, ε) were used to detect the effects of C-peptide and high glucose concentrations on the phosphorylation as an indication of activation of different PKC isoforms. Culturing of HRTC in the presence of high glucose leads to an increase in PKCα/β phosphorylation in the absence or in the presence of C-peptide, regardless of C-peptide concentrations (Fig. 2A). PKCδ phosphorylation was slightly increased after exposure to 10 nM, but not 1 nM of C-peptide (Fig. 2B). Exposure to 1 nM, but not 10 nM of C-peptide increased PKCε phosphorylation regardless of glucose concentrations (Fig. 2C).

### Physiological concentrations of C-peptide leads to ERK1/2 phosphorylation and nuclear localization

**Figure 3:**
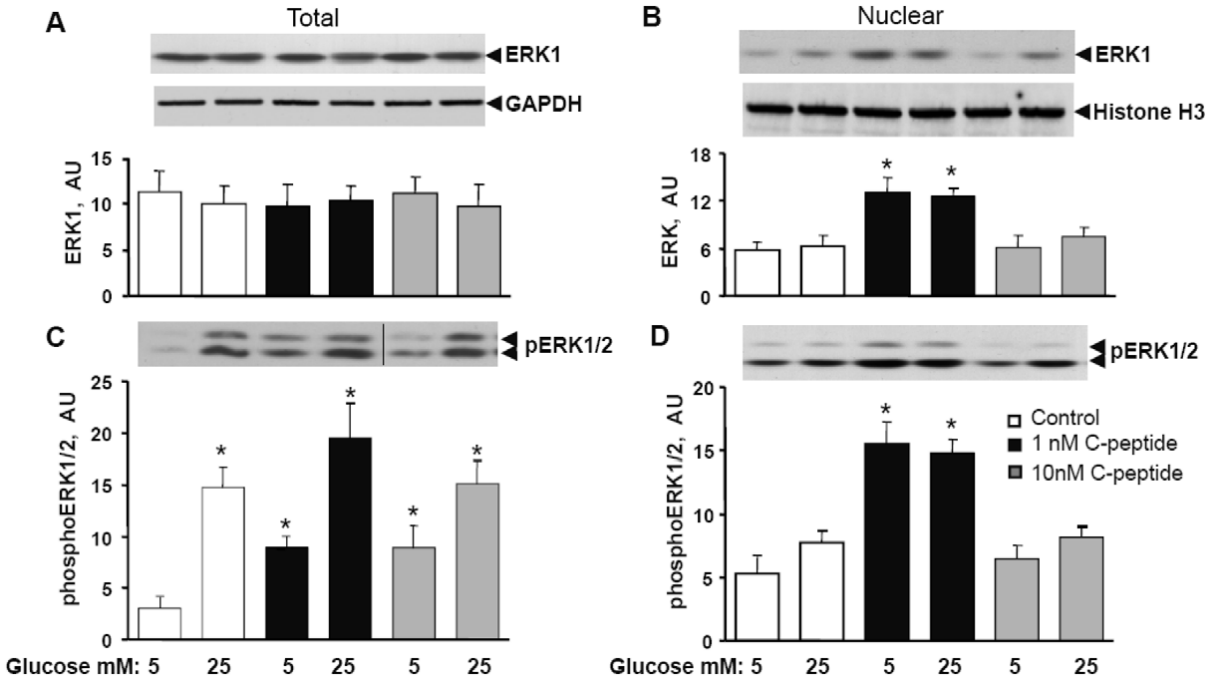
C-peptide in concentration of 1 nM stimulates ERK transocation into the nucleus and the kinase activatory phosphorylation. Cells were cultured as described in “Materials and Methods”. Total cell lysate and nuclear extracts were subject to Western blot analysis to determine ERK1/2 abundance and phosphorylation. A representative Western blot image is shown in the upper panel of each graph. **A.** The total ERK1 expression. **B.** ERK1 nuclear abundance . **C.** Total ERK1/2 phosphorylation. **D.** Nuclear ERK1/2 phosphorylation . GAPDH and histone H3 proteins were used as loading controls for total cell lysate and nuclear extracts, respectively. Results are means ± SE for 6 independent experiments. * P<0.05 versus 5 mM glucose without C-peptide.

The total ERK1 expression in HRTC cells was not affected by exposure to C-peptide or hyperglycaemia. (Fig. 3A). However, the ERK1 nuclear abundance was increased after exposure to 1 nM of but not 10 nM C-peptide (Fig. 3B). High glucose concentrations did not alter the effect of 1 nM C-peptide. Exposure to high glucose leads to an increase in phosphorylation of total ERK1/2, independent of the presence of C-peptide (Fig. 3C). At 5 mM glucose concentration both 1 nM and 10 nM C-peptide significantly stimulates total ERK1/2 phosphorylation (Fig. 3C). The effect of C-peptide on total ERK1/2 phosphorylation was not additive to the effect of hyperglycemia. However, only exposure to 1 nM of C-peptide increased nuclear ERK1/2 phosphorylation both in normal- and hyperglycemic conditions (Fig. 3D), reflecting increased nuclear ERK abundance (Fig. 3B).

### Effects of long term C-peptide and glucose exposure on ZEB DNA binding in HRTC

**Figure 4:**
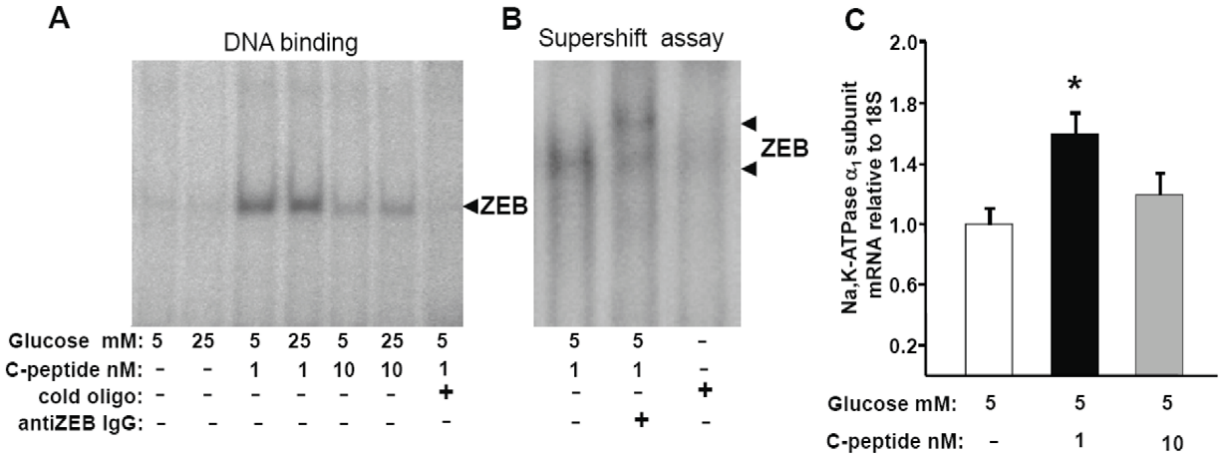
C-peptide induced transcription factor ZEB DNA binding and Na,K-ATPase α_1_-subunit mRNA expression. **A.** Exposure to 1 nM of C-peptide increased DNA binding activity of ZEB detected by EMSA. **B.** The electrophoretic mobility supershift assay with specific antibodies against ZEB. A–B. Experiments were performed at least 5 times and representative images are shown. **C.** mRNA expression of Na,K-ATPase α_1_-subunit. Results are means ± SE for 6 independent experiments. * P<0.05 versus 5 mM glucose without C-peptide.

In parallel with the C-peptide-induced increase Na, K, ATPase α_1_-subunit expression and ouabain-sensitive ^86^Rb^+^ uptake, exposure to 1 nM of C-peptide increased DNA binding activity of ZEB (AREB6) (Fig. 4A), a transcription factor involved in regulation of Na,K-ATPase α_1_-subunit expression [25]. In unstimulated cells basal ZEB DNA binding activity was nearly undetectable. ZEB DNA binding activity was only slightly increased by exposure to 10 nM C-peptide. The effect of C-peptide on ZEB DNA binding was unaffected by glucose concentration. The specificity of C-peptide induced ZEB DNA binding in HRTC was confirmed by electrophoretic mobility supershift assay utilizing specific antibodies against ZEB (Fig. 4B). The C-peptide-induced ZEB DNA binding activity was concomitant with increased Na,K-ATPase α_1_-subunit mRNA expression (Fig. 4C).

### C-peptide-induced expression of the Na,K-ATPase in HRTC is PKC- and ERK1/2 -dependent

**Figure 5:**
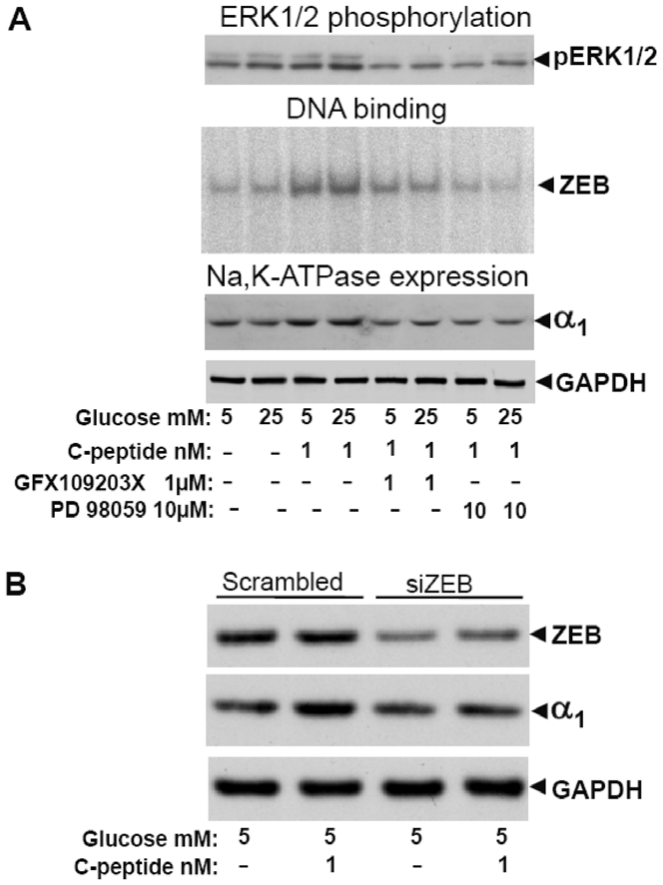
Na,K-ATPase expression regulates via PKC , ERK1/2 and ZEB activation. **A.** Cells were cultured, incubated with kinase inhibitors and analyzed as described in “Materials and Methods”. Experiments were performed at least 5 times and representative Western blot or EMSA images are shown. GAPDH protein was used as a loading control for total cell lysate. **B.** Effect of siRNA- mediated silencing of ZEB on ZEB and Na,K-ATPase α_1_-subunit protein expression in HRTC. GAPDH protein was used as a loading control. Experiments were performed at least 4 times and representative images are shown.

Effects of 1 nM C-peptide on ERK1/2 phosphorylation, ZEB DNA binding activity and Na,K-ATPase α_1_-subunit expression in HRTC were abolished by overnight incubation with a PKC inhibitor (1 µM GF109203X) or MEK1/2 inhibitor (10 µM PD98059), suggesting involvement of conventional and novel PKCs and ERK1/2 in regulation of C-peptide induced sodium pump expression (Fig. 5A).

### ZEB silencing in HRTC

Gene silencing of ZEB reduced protein abundance (Fig. 5B) by 60% (p<0.05), followed by a markedly reduced effect of 1 nM C-peptide on increase in Na,K-ATPase α_1_-subunit expression. Growth and gross morphology as assessed by light microscopy of HRTC with depleted ZEB expression were similar to those in cells transfected with siRNA against a scrambled sequence.

## Discussion

In recent years much new information has been presented indicating that C-peptide may be a bioactive endogenous hormone in its own right. Specifically, several reports have indicated that C-peptide elicits activation of Na,K-ATPase, an enzyme of crucial importance for cellular sodium and potassium ion homeostasis and membrane potential [2], [3], [12], [26]. Previously we have reported an acute stimulatory effect of the physiological concentrations of C-peptide on the sodium pump activity in HRTC [12]. In the present study we evaluated the long-term effect of C-peptide on Na,K-ATPase under normo- and hyperglycemic conditions in cells of human origin, and determined the molecular pathways of C-peptide action. Our results indicate that physiological concentrations of C-peptide stimulate Na,K-ATPase expression in HRTC by activation of a PKC- and MAP kinase-dependent pathway. Importantly, a scrambled sequence of the amino acids constituting human C-peptide was without effect on Na,K-ATPase expression, activity and cell signaling events. The stimulatory effect of C-peptide on sodium pump expression and activity overcomes the hyperglycemia-induced sodium pump downregulation. These findings are of particular interest since they are in agreement with *in vivo* studies in BB/W rats showing improved Na,K-ATPase levels in the sciatic nerve after exposure to physiological levels of C-peptide [3]. One week C-peptide infusion prevents type 1 diabetes-induced decrease of renal Na,K-ATPase α_1_-subunit in rats [2]. The observations also underscore the potential role of C-peptide as a therapeutic modality in patients with type 1 diabetes and its complications, particularly with regards to peripheral neuropathy [27].

Most of the changes in neuronal, vascular, muscular and renal Na,K-ATPase functions in the diabetic state have been linked to hyperglycemia via activation of PKC and a subsequent decrease in Na,K-ATPase activity due to phosphorylation of the alpha-subunit [28]. PKC activation elicits phosphorylation of the Na,K-ATPase α_1_-subunit and promotes endocytosis of the sodium pump units [29]. Indeed, in diabetic animal models, PKC activity is increased in retina, glomeruli, aorta, skeletal muscle, and heart, concomitant with decrease in Na,K-ATPase activity [30], [31], [32], [33]. Inhibition of PKCβ prevents the hyperglycemia-induced decrease in Na,K-ATPase activity in retina [15]. However, neither strict glycemic control nor restoration of normal glycemia and PKC activity [17], [34] can completely restore Na,K-ATPase activity.

We found that in human renal tubular cells hyperglycemia causes a decrease in Na,K-ATPase abundance and activity in basolateral membrane in parallel with activation of conventional PKCα and β. Conventional PKCs have been implicated in regulation of endocytosis of the sodium pump [20], [29]. However, only stimulation with physiological concentrations of C-peptide leads to activation of atypical PKCε. PKCε, together with another atypical PKC isoform, PKCδ, is involved in C-peptide-induced stimulation of ERK1/2 [8]. Thus, the present paper provides evidence to suggest that in HRTC, distinct pools and isoforms of PKCs are independently involved in hyperglycemia- and C-peptide-induced signaling.

Hyperglycemia activates the MAP kinase signaling pathway in number of cell types and tissues [28]. In HRTC high glucose concentrations stimulate total ERK1/2 phosphorylation C-peptide stimulation. However, only a physiological concentration of C-peptide promotes ERK nuclear accumulation and phosphorylation independently from glycemic status. Taken together, our findings provide evidence to suggest a pathway for chronic C-peptide signaling via PKCε activation, ERK1/2 nuclear accumulation and activation of nuclear transcription factors.

To explore the molecular mechanisms by which C-peptide and glycemic status regulates kidney cell Na,K-ATPase isoform expression, we determined transcription factor activity by using a DNA-binding assay. ZEB (AREB6) is expressed in multiple tissue/cell types and has been identified as a specific transcription factor regulating the Na,K-ATPase α_1_-subunit gene [25]. ZEB has a consensus sequence located in the promoter of the rat and human α_1_-subunit gene [23]. In rats, ZEB DNA binding activity and α_1_-subunit mRNA and protein expression was elevated after high-fat diet intervention, preceding the development of hyperinsulinemea and insulin resistance [35]. Despite these reported earlier correlative observations, the molecular mechanism regulating the genes encoding Na,K-ATPase is incompletely understood. We found that C-peptide increases ZEB DNA binding in HRTC, most profoundly in response to C-peptide within physiological concentration range. Importantly, this effect is independent from hyperglycemia. C-peptide-induced increase in ZEB DNA binding and Na,K-ATPase α_1_-subunit expression was abolished by PKC and MAP kinase inhibitors, which is in agreement with data concerning C-peptide-induced specific PKCε and ERK1/2 activation. Moreover, partial gene silencing of ZEB markedly reduced effect of C-peptide on increase in Na,K-ATPase α_1_-subunit expression. However, the direct effects of PKCε and ERK1/2 on ZEB activation require further elucidation.

**Figure 6:**
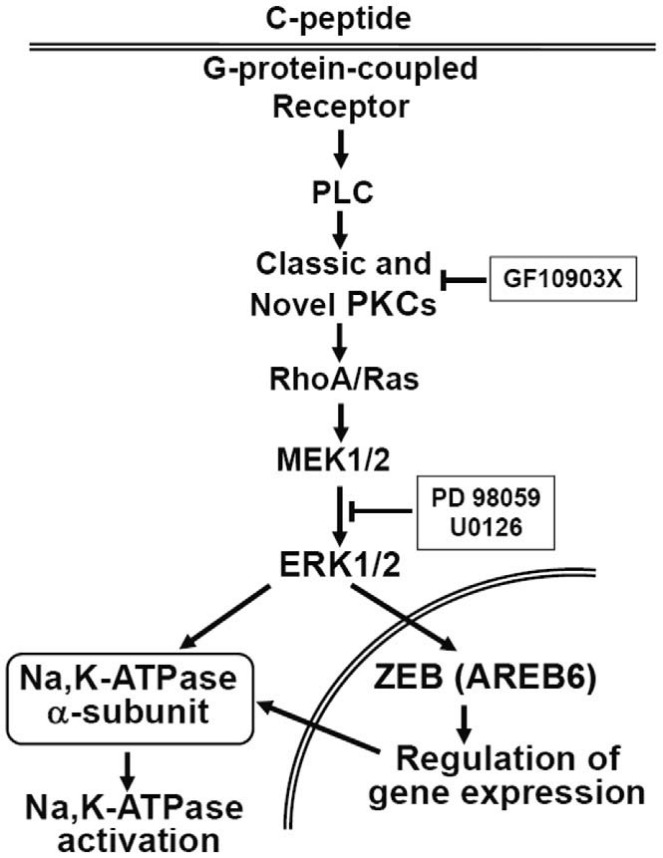
Schematic representations of intracellular signaling pathways for regulation of the sodium pump activity by C- peptide in human renal tubular cells. C- peptide binds specifically to a membrane structure, most likely a G-protein coupled receptor, with subsequent activation of PLC, isoforms of both classic and novel PKC, Rho A, MEK1/2 and ERK1/2. The latter elicits activation of ZEB (AREB6) and regulation of the gene expression for Na,K-ATPase α_1_-subunit.

We have previously shown that C-peptide stimulates Na,K-ATPase activity in HRTC acutely via translocation of α_1_- and β_1_-subunits to the basolateral membrane from an endosomal compartment by PKC- and ERK1/2 dependent mechanism [12]. Here we provide evidence to show that physiological concentrations of C-peptide stimulates sodium pump expression via activation of ZEB, a transcription factor that has not been previously implicated in C-peptide-mediated signaling. Our findings suggest that despite activation of ERK1/2 and PKC by hyperglycemia, distinct pools of PKCs and ERK1/2 are involved in the regulation of Na,K-ATPase expression and activity by C-peptide. Taken together, our findings suggest that PKCs and MAP kinases are essential for C-peptide-stimulated Na,K-ATPase activation (Fig. 6). These data support the growing body of evidence for the involvement of ERK1/2 in acute and long term Na,K-ATPase activation in different tissues.

## Materials and Methods

### Antibodies and reagents

Specific anti-α_1_-subunit monoclonal antibodies were obtained from Dr. M. Caplan (Yale University, New Haven, CT). Rabbit polyclonal anti-phospho PKC α/β, δ, ε and histone H3 were from Cell Signaling Technology, Inc. (Beverly, MA) Monoclonal anti-phospho-ERK1/2 (P-Thr ^202^/Tyr^204^) and polyclonal anti-ERK1 were from New England BioLabs (Beverly, MA). Rabbit antibodies raised against zinc finger/homeodomain protein (ZEB), GLUT2 and GAPDH were purchased from Santa Cruz Biotechnology (Santa Cruz, CA). Kinase inhibitors PD98059 and GF109203X were from Calbiochem (La Jolla, CA). Cell culture media and reagents were obtained from Invitrogen, Sweden. Dimethylsulfoxide from Calbiochem was used as a solvent for protein kinase inhibitors. All other reagents were of analytical grade. Human recombinant C-peptide was obtained from Schwarz Pharma (Monheim, Germany). Scrambled C-peptide (the same amino acid residues as in C-peptide but in random order) was from Sigma Genosys (Cambridge, UK). Horseradish peroxidase-conjugated goat anti-rabbit and anti-mouse immunoglobulin G was obtained from Bio-Rad Laboratories (Hercules, CA). Reagents for enhanced chemiluminescence were obtained from Amersham (Arlington Heights, IL). All other reagents were of analytical grade (Sigma).

### Cell culture

Human renal tubular cells (HRTC) were cultured from the unaffected outer cortex of renal tissue obtained from non-diabetic patients undergoing elective nephrectomy for renal carcinoma. Tissue collection was undertaken with the written informed consent of the subject and approval by the Karolinska Institutet Ethics Committee. The cells were cultured in RPMI 1640 supplemented with 10% fetal calf serum, 2 mM L-glutamine, 5 mM glucose,10 mM HEPES, bensylpenicillin (100 U/ml) and streptomycin (100 µg/ml) and passaged at near confluence by trypsinization. Growing cells exhibited epithelial morphology with a central nucleus, a granular cytoplasm and cobblestone appearance on light microscopy. Cells from the second and third passages were used for experiments. Then cells were cultured for 5 days in 5 mM glucose and 20 mM mannitol, or in 25 mM glucose in absence or presence of 1 nM or 10 nM of C-peptide, as indicated. The cell culture medium with all additions was replaced daily.

### siRNA transfections in HRTC

HRTC cultured in 6-well plates were transfected using Lipofectamine 2000 reagent (Invitrogen). The medium was changed to antibiotic-free growth medium on day 1 of the HRTC culture protocol. On day 2 HRTC were transfected with individual siRNAs (1 µg/ml) using Lipofectamine 2000 in serum-free RPMI 1640 media. The siRNA sequence GGU AGA UGG UAA UGU AAU Att (Life Technologies) directed against human ZEB or a scrambled sequence were used. Cells were washed with PBS, and 2 ml of RPMI 1640 supplemented with 10% fetal calf serum, 2 mM L-glutamine, 5 mM glucose, 10 mM HEPES was then added to each well. Then cells were cultured for 5 days in 5 mM glucose and 20 mM mannitol, in absence or presence of 1 nM of C-peptide.

### Cell incubation

For total Na,K-ATPase and ERK1 expression and PKC phosphorylation analysis, cells were washed twice with ice-cold PBS, and harvested by scraping cells into ice-cold lysis buffer A (20 mM Tris pH 8.0, 135 mM NaCl, 1 mM MgCl_2_, 2.7 mM KCl, 10 mM Na_4_P_2_O_7_, 0.5 mM Na_3_VO_4_, 10 mM NaF, 1 µM okadaic acid, 1% Triton X-100, 10% v/v glycerol, 0.2 mM PMSF, 10 µg/ml leupeptin, and 10 µg/ml aprotinin). Homogenates were rotated for 60 min at 4°C and subjected to centrifugation (12,000×g for 10 min at 4°C). Protein concentration was determined using a bicinchoninic acid protein assay kit (Pierce Chemical Co., Rockford, IL). Lysates were kept at −80°C before subsequent Western blot analysis with appropriate antibodies.

### Measurement of ouabain-sensitive ^86^Rb^+^ uptake

The transport activity of Na,K-ATPase was measured by ouabain-sensitive ^86^Rb^+^ uptake under conditions of initial rate, as described [12]. The cells were grown on 6-well plates (Costar, Cambridge, MA) in 5 mM glucose and 20 mM mannitol, or in 25 mM glucose in absence or presence of 1 nM or 10 nM of C-peptide, as indicated. Before the measurement, cells were preincubated in 950 µl serum-free RPMI 1640 without or with ouabain (1 mM) for 15 min at 37°C. Thereafter the transport activity of Na,K-ATPase was determined after the addition of 50 µl of medium containing tracer amounts of ^86^RbCl (100 nCi/sample; Amersham, UK) for 15 min. Incubation was stopped by cooling on ice and dishes were washed three times with an ice-cold solution containing 150 mM choline-chloride, 1.2 mM MgSO_4_, 1.2 mM CaCl_2_, 2 mM BaCl_2_, and 5 mM HEPES, pH 7.4. Cells were lysed in 750 µl lysis buffer A, and the radioactivity was measured by liquid scintillation. Protein content was determined in parallel by using the bicinchoninic acid assay (Pierce, Rockford, IL). The ouabain-sensitive ^86^Rb^+^ uptake was calculated as the difference between the mean values measured in triplicate samples incubated without or with 0.2 mM ouabain and was expressed as % of control. Basal ouabain-sensitive ^86^Rb^+^ uptake was 16.5±2.3 picomoles Rb^+^ per microgram of protein per minute.

### Preparation of basolateral membrane from HRTC

After treatment cells were washed twice with ice-cold PBS, and harvested by scraping cells into 500 µl of ice-cold buffer (12 mM HEPES, 300 mM mannitol, pH 7.6 (Tris 1 M), 0.5 mM Na_3_VO_4_, 0.2 mM PMSF, 5 µg/ml leupeptin, 5 µg/ml aprotinin and 1 µM microcystin). Cells were homogenized by Pellet Pestle twice for 1 min, and passing through insulin syringe with 21G needle 10–12 times into an Eppendorf tube. Homogenates were pre-cleaned by centrifugation at 2,500×g for 15 min. Supernatants were collected. Pellets were resuspended in 500 µl of homogenization buffer, and spun down at 2,500×g for 15 min. Supernatants were collected and transferred to the tubes, containing the first 500 µl, and centrifuged at 20,000×g for 20 min, +4° C on TLA 100.2 Beckman rotor. Basolateral membranes (BLM) were further purified [12], using a Percoll gradient. The yellow layer of pellet was resuspended again in the supernatant (carefully removed from the brown pellet containing mitochondria and cell ghosts) and centrifuged at 48,000×*g* for 30 min. The supernatant was discarded, and the pellet was resuspended in 1 ml of buffer (300 mM mannitol and 12 mM HEPES, pH 7.6, adjusted with Tris) by gentle pipetting. To form a Percoll gradient, 0.19 g of undiluted Percoll (Pharmacia Biotech Inc.) was added to a 1-ml suspension (0.8–1.0 mg of protein). The suspension was gently mixed and centrifuged at 48,000×*g* for 30 min, and the ring of BLM, light endosomal fraction (the top 1/3 part of the tube), and pellets were collected and frozen at −20°C. Protein content was determined by using the bicinchoninic acid assay (Pierce).

### Determination of Na,K-ATPase activity

Na,K-ATPase activity was measured at maximum velocity (V_max_) conditions in BLM fractions, essentially as described [36]. Aliquots of BLM fractions (protein content, 2–3 µg) were transferred to the Na,K-ATPase assay medium (final volume 100 µl), containing 50 mM NaCl, 5 mm KCl, 10 mM MgCl_2_, 1 mM EGTA, 50 mm Tris-HCl, 10 mM Na_2_ATP, and γ-^32^P-ATP (NEN Life Science Products; specific activity, 3000 Ci/mmol) in tracer amounts (3.3 nCi/µl), at 4°C. The samples were then incubated at 37°C for 15 min. The reaction was terminated by rapid cooling to 4°C and addition of a mixture of trichloroacetic acid/charcoal (5%/10%). After separating the charcoal phase (12,000×g for 5 min) containing the nonhydrolyzed nucleotide, the ^32^Pi liberated in the supernatant was counted. Na,K-ATPase activity was calculated as the difference between test samples (total ATPase activity) and samples assayed in a medium devoid of Na^+^ and K^+^ and in the presence of 2 mM ouabain (ouabain-insensitive ATPase activity). Protein determination was performed as described above.

### Nuclear extraction

The nuclear extraction procedure was essentially as described previously [37], with some modifications. Cells were grown in 10-cm Petri dishes, at the last day of the experiment were incubated overnight with 20 µM PD98059 (MEK1 inhibitor), 1 µM or 10 µM GF109203X (PKC inhibitor), as indicated and then washed with ice-cold PBS. Cells were scraped in 600 µl of PBS and transferred to microcentrifuge tubes. After centrifugation at 350 g for 30 s, cells were resuspended in 300 µl of buffer [10 mM HEPES-KOH, pH 7.9, 1.5 mM MgCl_2_, 10 mM KCl, 0.5 mM dithiothreitol (DTT), and 0.2 mM phenylmethylsulfonyl fluoride (PMSF)]. Cells were left to swell for 15 min. After centrifugation, the pellet, which contains the nuclear proteins, was resuspended with 20 µl of buffer (20 mM HEPES-KOH, pH 7.9, 1.5 mM MgCl_2_, 420 mM NaCl, 25% glycerol, 0.2 mM EDTA, 0.5 mM DTT, and 0.2 mM PMSF). The nuclear proteins were left to diffuse into the buffer for 30 min. The supernatant, with the nuclear proteins, was collected in microcentrifuge tubes supplemented with 50 µl of low-saline buffer (10 mM HEPES-KOH, pH 7.9, 1.5 mM MgCl_2_, 0.5 mM DTT, and 0.2 mM PMSF). Protein concentration of the resulting supernatant was determined using a bicinchoninic acid assay (Pierce).

### Electrophoretic mobility shift assay

ZEB DNA binding activity was determined by an electrophoretic gel mobility shift assay (EMSA) as described previously [34]. Oligonucleotides for the consensus binding sites for zinc finger/homeodomain protein (ZEB) transcription factor were purchased from Santa Cruz Biotechnology (Santa Cruz, CA). Oligonucleotides were end-labeled with T4 polynucleotide kinase (Santa Cruz) and 2 µCi of [γ-^32^P]ATP. Nuclear extracts (1.5–2.5 µg) were incubated for 20 min in reaction mixture containing 2 µg poly(dI-dC), 0.5 mM DTT, 25 mM Tris-HEPES, pH 7.5, 60 mM KCl, 1 mM EDTA, and 12% glycerol (15 min on ice and 5 min at room temperature). As a negative control, unlabeled competitor oligonucleotide was incubated with stimulated cell nuclear extract before the addition of labeled probe. ^32^P-labeled probes (0.5 ng) were incubated with the protein-mixture complex for 30 min at room temperature. Thereafter, 20 µl aliquots were loaded on a nondenaturing 5% polyacrylamide (30% acrylamide/bisacrylamide) gel buffered with Tris-borate-EDTA (10 mM Tris, 90 mM boric acid, 1 mM EDTA) and subjected to electrophoresis at 40 mA for 1.5 h at room temperature. The dried gels were analyzed using a phosphoimager (Fuji model BAS-1800II). For supershift analysis of ZEB, nuclear extracts were incubated for 1 h with ZEB antibodies (Santa Cruz). To exclude nonspecific binding, extracts were incubated with unlabeled ZEB oligo. The mixture was then incubated with radioactively labeled oligo for 30 min at room temperature. Samples were loaded as described above.

### Real time PCR

Quantification of the mRNA expression of Na,K-ATPase α_1_-subunits from HRTC was performed using quantitative real-time PCR with the ABI PRISM 7000 Sequence Detector System and fluorescence-based SYBR Green technology (Applied Biosystems,Warrington, UK). Total RNA was prepared from the tissue samples using TRIzol reagent (Invitrogen, Carlsbad, CA) according to the manufacturer's protocol. Synthesis of cDNA was performed with random hexamer primers using SuperScript First Strand Synthesis System (Invitrogen). Real-time PCR reactions contained 400 nm of each PCR primer and 1× SYBR Green PCR Master Mix (Applied Biosystems). The forward (F) and reverse (R) primer sequences were as follows: for Atp1a1 (GenBank NM000707.7) GGCTGTCATCTTCCTCATTGG (F) and CGGTGGCCAGCAAACC (R). All samples were run in duplicate, and the relative quantities of different mRNA transcripts were calculated after normalization of the data against 18S, as endogenous controls, using relative quantification method.

### Western blot analysis

Aliquots of cell lysates (30 µg of protein) and fractions of basolateral membranes (10 µg of protein) were resuspended in Laemmli sample buffer. Proteins were then separated by SDS/PAGE, transferred to PVDF membranes, blocked with 7.5% non-fat milk, washed with TBST (10 mM Tris HCl, 100 mM NaCl, 0.02% Tween 20) and incubated with appropriate antibodies overnight at 4°C. Membranes were washed with TBST and incubated with an appropriate secondary antibody. Proteins were visualized by enhanced chemiluminescence and quantified by densitometry.

### Statistics

Data are presented as mean ± SE. Comparisons between groups were performed using Student's *t*-test. For multiple comparisons, one-way ANOVA with Scheffe's correction was used. Significance was established at *P*<0.05.
